# Imaging the development of chronic Chagas disease after oral transmission

**DOI:** 10.1038/s41598-018-29564-7

**Published:** 2018-07-26

**Authors:** Michael D. Lewis, Amanda F. Francisco, Shiromani Jayawardhana, Harry Langston, Martin C. Taylor, John M. Kelly

**Affiliations:** Department of Pathogen Molecular Biology, Faculty of Infectious and Tropical Diseases, London School of Hygiene and Tropical Medicine, Keppel Street, London, WC1E 7HT United Kingdom

## Abstract

Chagas disease is a zoonosis caused by the protozoan parasite *Trypanosoma cruzi*. Transmission cycles are maintained by haematophagous triatomine bug vectors that carry infective *T. cruzi* in their faeces. Most human infections are acquired by contamination of mucosal membranes with triatomine faeces after being bitten, however, *T. cruzi* can be transmitted by several other routes. Oral transmission is an increasingly important aspect of Chagas disease epidemiology, typically involving food or drink products contaminated with triatomines. This has recently caused numerous outbreaks and been linked to unusually severe acute infections. The long-term impact of oral transmission on infection dynamics and disease pathogenesis is unclear. We used highly sensitive bioluminescence imaging and quantitative histopathology to study orally transmitted *T. cruzi* infections in mice. Both metacyclic and bloodform trypomastigotes were infectious via the oral cavity, but only metacyclics led to established infections by intra-gastric gavage. Mice displayed only mild acute symptoms but later developed significantly increased myocardial collagen content (*p* = 0.017), indicative of fibrosis. Gastrointestinal tissues and skin were the principal chronic infection reservoirs. Chronic phase parasite load profiles, tissue distribution and myocardial fibrosis severity were comparable to needle-injected controls. Thus, the oral route neither exacerbates nor ameliorates experimental Chagas disease.

## Introduction

Chagas disease (American trypanosomiasis) is caused by infection with *Trypanosoma cruzi*, a protozoan parasite. Approximately 6 million people are infected and the disease causes ~13,000 deaths annually and a large morbidity burden in affected populations^1^. Chagas disease is endemic in most of Latin America and is becoming an increasingly important global public health problem as a result of migration^2,3^. *T. cruzi* has a complex life cycle^4,5^, as part of which it is transmitted between mammalian hosts, including humans, by blood-feeding triatomine bugs. Epimastigote parasites replicate in the insect’s midgut before migrating to the hindgut, where they can transform into non-dividing metacyclic trypomastigotes (MTs), which are infectious to mammals. When a triatomine takes a blood meal it frequently deposits faeces containing MTs onto its host. Thereafter, the MT parasites can contaminate the bite site or mucosal tissues, leading to infection. MTs actively invade host cells and transform into a third developmental form, the amastigote, which replicates in the host cell cytosol. After several rounds of replication, amastigotes make a developmental transition resulting in bloodstream trypomastigotes (BTs), which can either invade a new host cell, or complete the life cycle by transforming into epimastigotes if they are taken up by a triatomine bug as part of a blood meal.

Control programs have successfully reduced or interrupted vectorial transmission in many regions^6,7^. One consequence of this is a renewed focus on other sources of infection, including the congenital route and transmission from donors to transplant or transfusion recipients. The oral transmission route has garnered particular attention, not least because of recurring outbreaks involving parasite-contaminated food and drink products^8–15^. The number of acute Chagas disease cases attributed to oral infection has increased dramatically since 2000^1,16^. It is not clear whether this trend reflects better surveillance or genuine changes in transmission patterns driven by eco-epidemiological factors. The oral route is also important because consumption of triatomines by wild mammals is a key part of natural *T. cruzi* transmission cycles^17^. Oral vaccination of domesticated animal reservoirs, e.g. dogs, is also envisaged as a potentially valuable disease control measure^18^.

The effect that transmission routes have on Chagas disease clinical outcomes is poorly understood. Vector transmitted infections are rarely diagnosed in the acute phase and even amongst those that are, the symptoms are generally mild, with fewer than 5% proving fatal; the remainder progress to life-long chronic infections^19^. In contrast, some oral outbreaks have been associated with unusually severe symptoms and high fatality rates, often >20%^16^, raising concerns that oral transmission is particularly dangerous. Experimental studies have reproduced features of severe acute Chagas disease in mice after oral inoculation^20,21^.

It is not known whether reports of increased severity of orally-acquired infections can be explained by the route of parasite entry or other co-varying factors. Furthermore, the longer term impact of oral transmission on chronic disease progression and severity has not been studied. We recently developed an enhanced sensitivity real-time imaging method to assess chronic *T. cruzi* infections^22,23^, which has enabled us to address these questions.

## Results

### Oral transmission efficiency and course of infection

We used a high sensitivity *T. cruzi in vivo* imaging model to track infections in mice after inoculation of parasites via intra-peritoneal (i.p.) injection, intra-gastric gavage, or buccal deposition (Table 1). Gavage of MTs, the infectious form present in insect vectors, led to established infections in 25% of mice (n = 16), compared to 92% in the needle-injected controls (n = 13). The MT inoculum was not transmissible via the oral cavity (n = 6). When the number of parasites in the inoculum was increased from 10^4^ to 10^5^, infectivity was boosted to 67% via gavage (n = 3) and 100% via oral cavity (n = 3). We were unable to infect mice with BTs via the stomach (n = 10). However, buccal inoculation of BTs led to established infections in 36% of mice (n = 11), compared to 100% in the needle-injected controls (n = 13). There were no fulminant infections and no disparities between animals infected via different routes with respect to weight, body condition or activity.

**Table 1:** Infectivity of *Trypansoma cruzi* in mice by oral routes.

Trypomastigote type	Route	Dose	Inoculated (n)	Infected (n)	Infectivity (%)
Metacyclic	Needle (i.p.)^a^	1 × 10^4^	13	12	92.3
Metacyclic	Oral (g)^b^	1 × 10^4^	16	4	25.0
Metacyclic	Oral (g)^b^	1 × 10^5^	3	2	66.7
Metacyclic	Oral (g)^b^	Either^d^	19	6	31.6
Metacyclic	Oral (b)^c^	1 × 10^4^	6	0	0.0
Metacyclic	Oral (b)^c^	1 × 10^5^	3	3	100
Metacyclic	Oral (b)^c^	Either ^d^	9	3	33.3
Blood	Needle (i.p.)^a^	1 × 10^4^	13	13	100
Blood	Oral (g)^b^	1 × 10^4^	10	0	0.0
Blood	Oral (b)^c^	1 × 10^4^	11	4	36.4
Blood	Oral (b)^c^	1 × 10^5^	3	0	0.0
Blood	Oral (b)^c^	Either^d^	14	4	28.6

^a^Intra-peritoneal; ^b^intra-gastric gavage; ^c^buccal administration; ^d^1 × 10^4^ and 1 × 10^5^ data combined.

**Figure 1:**
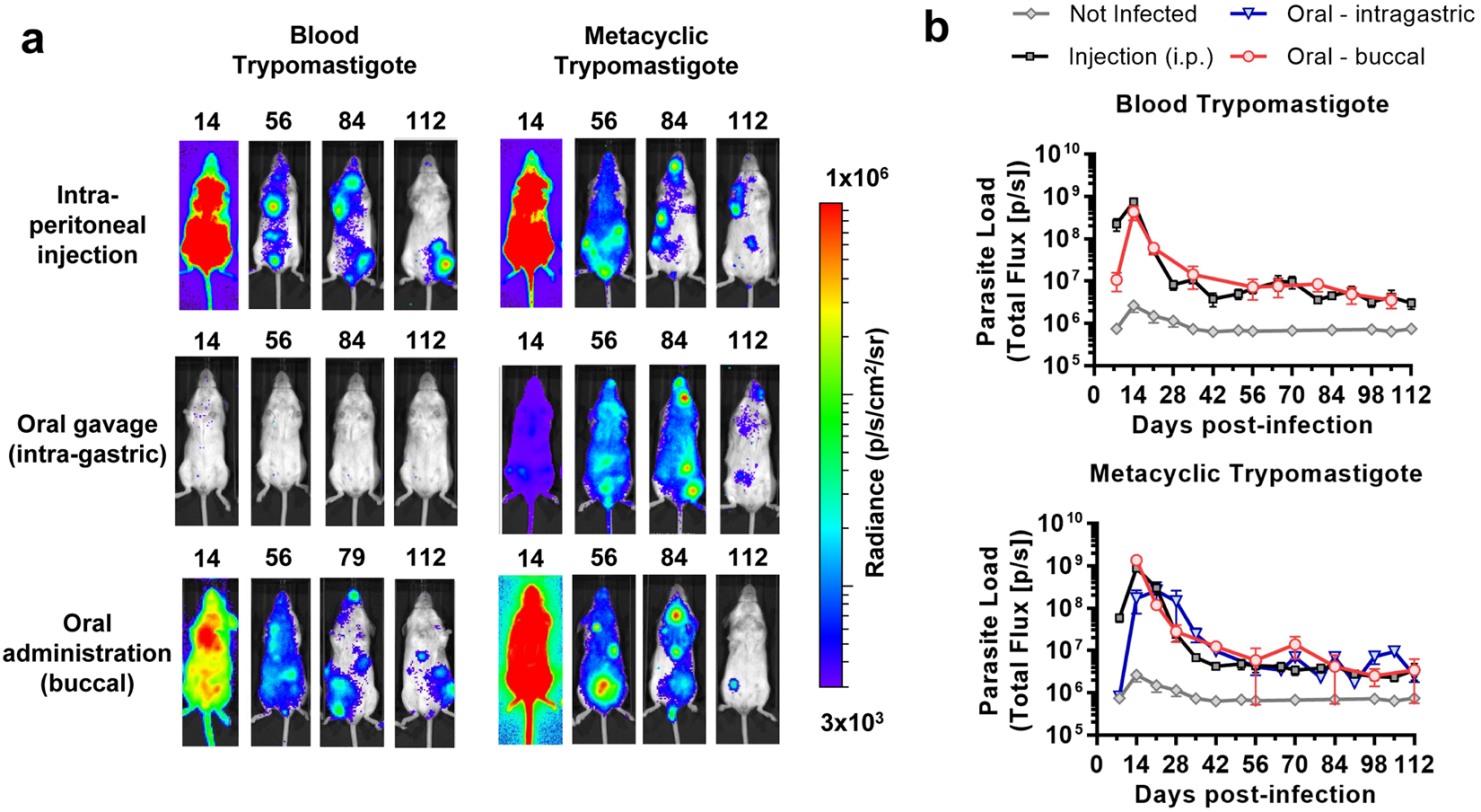
Serial evaluation of *T. cruzi* infection after oral transmission by *in vivo* bioluminescence imaging. (**a**) Serial ventral images of individual mice infected with blood or metacyclic trypomastigotes mouse via oral routes or needle injection (n = 6–16). Log-scale pseudocolour heat-maps show intensity of bioluminescence; minimum and maximum radiances are indicated. (**b**) Charts show mean bioluminescence ± SEM for mice from the experiment represented by images in (**a**) and non-infected control mice (n = 10) to show background luminescence. Mice that were inoculated with *T. cruzi* but did not develop an infection were excluded, including the entire intra-gastric blood trypomastigote gavage cohort.

**Figure 2:**
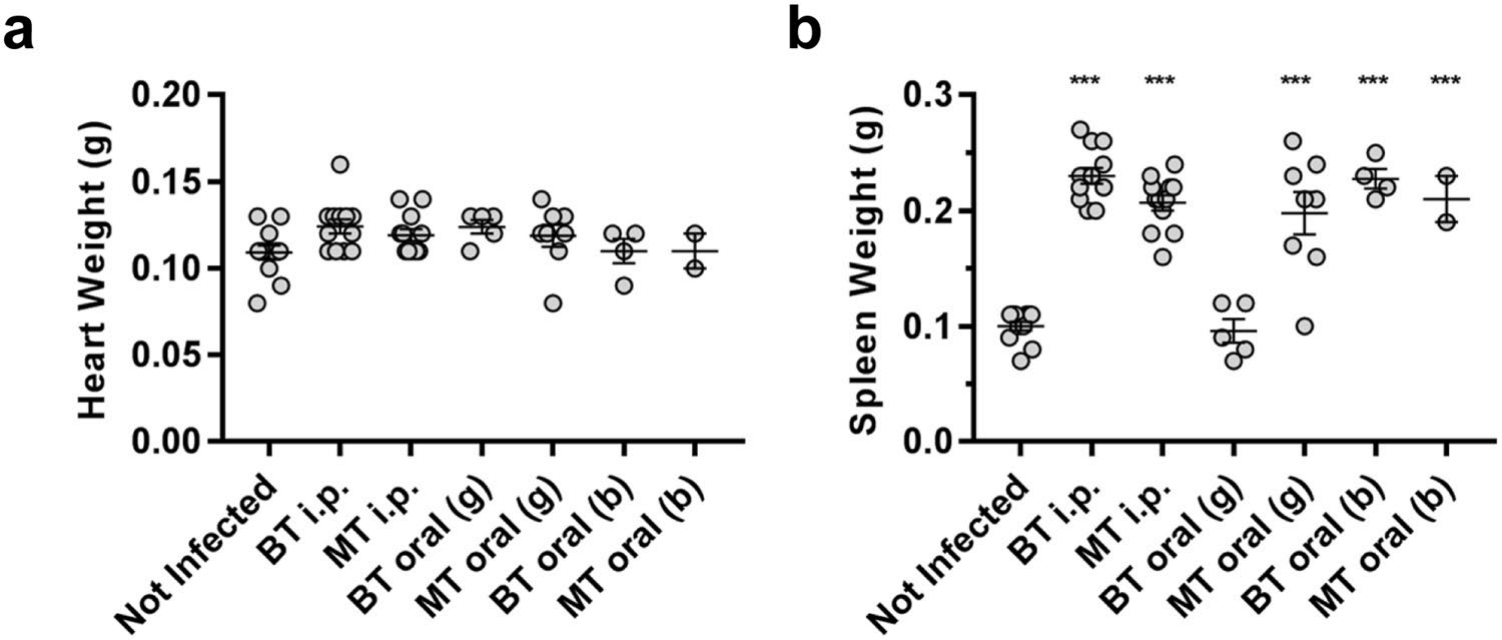
Heart and spleen weights in mice chronically infected with *T. cruzi* after oral transmission. Mice were infected with either metacyclic or bloodstream *T. cruzi* trypomastigotes (MT, BT) by i.p. injection, oral gavage (g) into the stomach or deposition in the oral cavity (b). Hearts (**a**) and spleens (**b**) from mice in which chronic infections were established were weighed at necropsy 4 months post-infection.

Serial bioluminescence imaging (Fig. 1) showed that those mice that did acquire orally-transmitted *T. cruzi* infections initially had lower average parasite loads compared to the needle-injected controls. The parasite load at the acute peak of infection, 14–21 days post-infection (dpi), was systemically distributed and quantitatively similar in all groups except the MTs intra-gastric gavage cohort, in which it was significantly lower than the i.p. injection controls (one way ANOVA, *p* = 0.01). The immune-mediated clearance phase proceeded similarly in all groups, involving an approximately 100-fold parasite load reduction by 56 dpi. Thereafter, chronic infections were characterised by spatiotemporally dynamic bioluminescent foci, with parasite loads fluctuating around similar means. Heart weights did not vary significantly between groups, but spleen weights were significantly, and equivalently increased in all infected groups, apart from the BT intra-gastric gavage cohort, in which no infections were established (Fig. 2).

We conclude that oral transmission of the TcVI-CL Brener *T. cruzi* strain is relatively inefficient. When infections are established, there are reduced parasite loads in the early acute stage, but the long-term infection dynamics are equivalent to needle-injected controls.

### Tissue tropism after oral transmission

**Figure 3:**
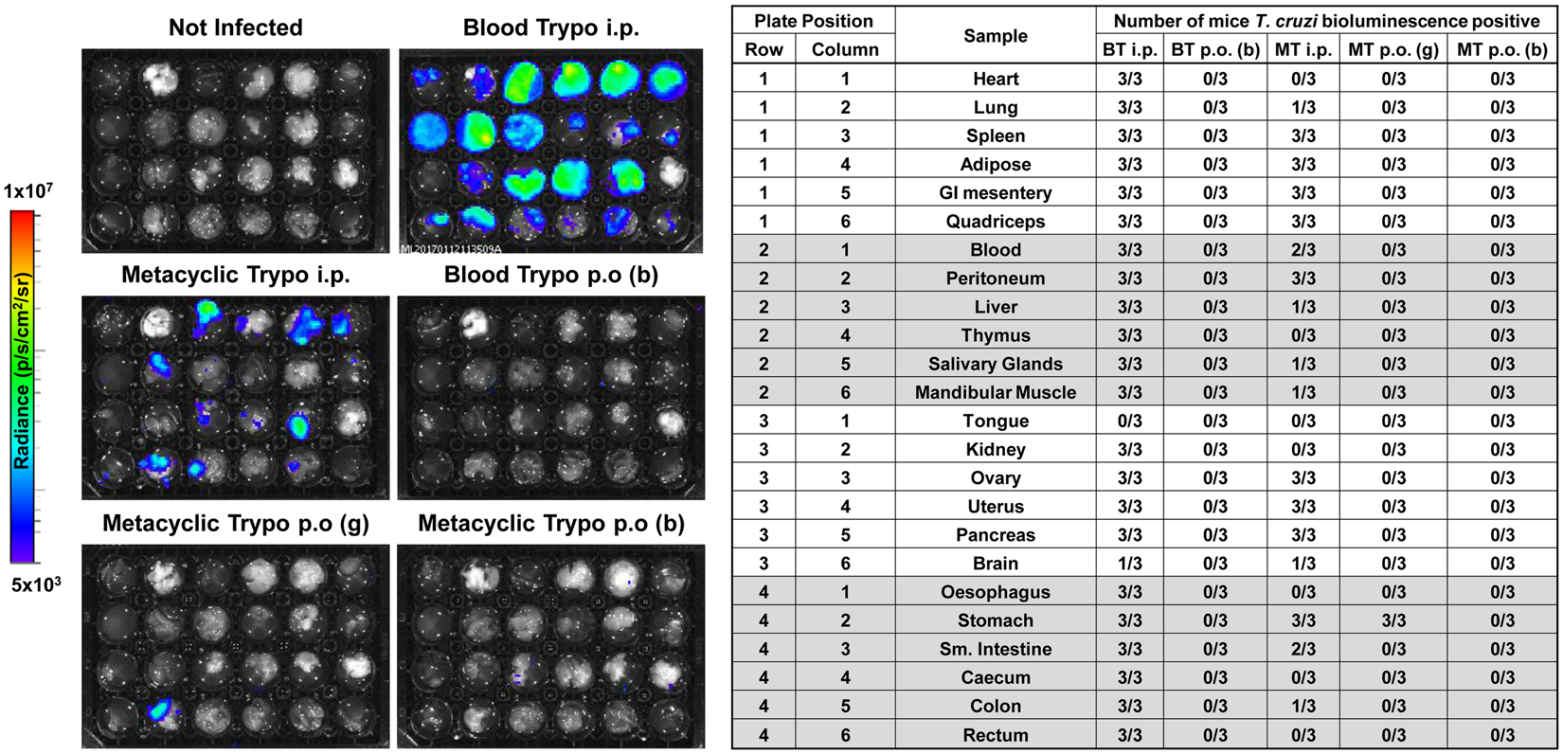
*Ex vivo* tissue bioluminescence imaging of mice acutely infected with *T. cruzi* after oral transmission. Mice were infected with 10^5^ metacyclic or bloodstream *T. cruzi* trypomastigotes (MT, BT) by i.p. injection, oral gavage [p.o. (g)] into the stomach or deposition in the oral cavity [p.o. (b)]. Parasite distribution was assessed by bioluminescence signal in organs and tissue samples from mice at 6 days post-infection. Log-scale pseudocolour heat-maps show intensity of bioluminescence; minimum and maximum radiances are indicated. Samples were arranged as indicated in the table. Images are representative of three independent replicates with the table showing the overall assessment of *T. cruzi* presence vs. absence in each sample.

During the acute infection phase, *T. cruzi* is able to parasitize highly diverse cell types and tissues^24^, but the actual distribution of parasites could be affected by the route of entry. We therefore tested the hypothesis that parasite distribution is affected by the transmission route. To establish the early targets of *T. cruzi* infection, organs and tissues were excised and imaged at 6 dpi (Fig. 3). Mice that were needle-injected with either MTs or BTs already had broadly disseminated infections by this time-point, with the spleen, GI tract and adipose-rich tissues having the highest parasite loads. In mice that received MTs by intra-gastric gavage, parasites were only detected in the stomach. Infections were undetectable in any organ/tissue from animals inoculated via the oral cavity with BTs or MTs, suggesting that very few parasites in the inoculum were productively transmitted.

**Figure 4:**
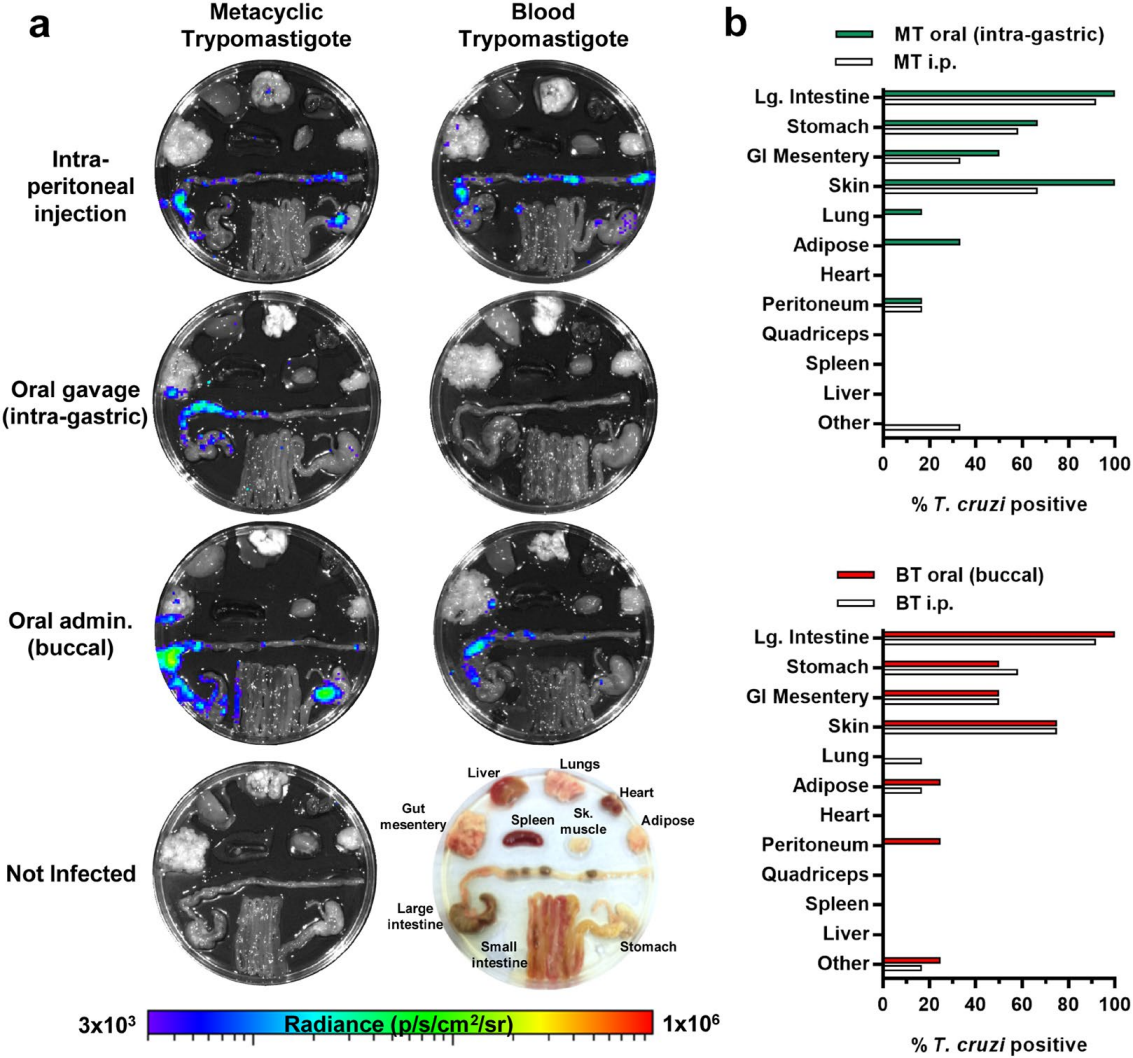
Tissue parasite distribution in mice chronically infected with *T. cruzi* after oral transmission. (**a**) Representative *ex vivo* images of tissue-specific parasite bioluminescence at 4 months post-infection. Images show infection distributions and intensities for adipose, gut mesenteric tissue, heart, lung, large intestine, liver, skeletal muscle, spleen, small intestine and stomach. Log-scale pseudocolour heat-maps show intensity of bioluminescence; minimum and maximum radiances are indicated. Tissues and organs are arranged as shown (bottom right). (**b**) Charts show the proportion of animals that had above threshold bioluminescence in the indicated organs and tissue samples. The category “Other” refers to bioluminescence signals in the animal carcass that could not be unambiguously assigned to a specific organ. MT oral (intra-gastric) n = 6, MT i.p. n = 12, BT oral (buccal) n = 4, BT i.p. n = 12.

To establish the long-term reservoirs of *T. cruzi* infection in the chronic phase, organs and tissues were excised and imaged at 4 months post-infection (Fig. 4 and Supplementary Fig. 1). Consistent with the *in vivo* imaging data, mice that were gavaged with BTs showed no evidence of infection in any tissues. In all other experimental groups, the primary sites of chronic parasite persistence were the large intestine, stomach, GI mesenteries and the skin. Other notable sites that were *T. cruzi* positive in only a minority of animals included lung, adipose and peritoneum. The level of dissemination in chronic infections, gauged by calculating the number of parasite positive sites per animal, was higher on average after oral transmission than after i.p. injection, although this was not statistically significant (Supplementary Fig. 2). Otherwise, the chronic tissue distributions in orally infected animals were broadly comparable with needle-injected controls (Fig. 4b).

### Cardiac pathology after oral transmission

**Figure 5:**
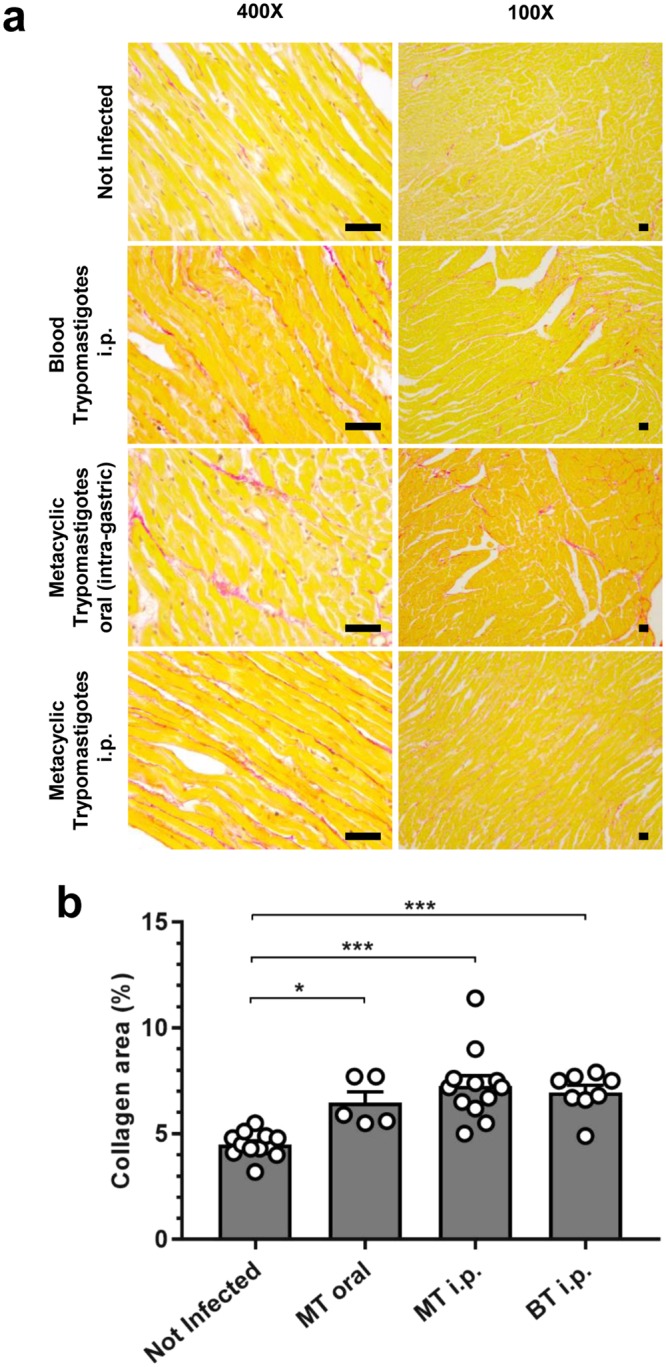
Cardiac fibrosis in mice chronically infected with *T. cruzi* after oral transmission. (**a**) Representative myocardial sections stained with picro-sirius red, magnification 400X and 100X , scale bar = 100 µm. (**b**) Quantification of collagen content (% red area in stained sections) as a marker of cardiac fibrosis severity. Data are the means ± SEM, pooled from three experiments. Not infected n = 12, MT oral n = 5, MT i.p. n = 12, BT i.p. n = 8. Asterisks indicate *p*-values for comparisons with the not infected control group in a one-way ANOVA test (^*^p < 0.05; ^***^p < 0.001).

Fibrosis of the myocardium is central to the pathogenesis of cardiac Chagas disease^25,26^. We used quantitative histopathological analysis of myocardial collagen content and found a significant increase in mice that had been infected via the oral route compared to uninfected animals (Fig. 5). The needle-injected controls also had significant fibrosis. There was no significant difference in severity between the oral infection and needle-injection cohorts. The extent of myocardial fibrosis in these animals was also similar to our findings for subcutaneously injected mice^23^. We conclude that orally acquired *T. cruzi* infection causes chronic cardiomyopathy, although this route of transmission does not exacerbate the condition in this model.

## Discussion

*Trypanosoma cruzi* is unusual, though not unique, amongst protozoan parasites in its ability to establish a systemic infection in its host after transmission via an oral route^27^. Most human cases involve drinks contaminated with insect-derived MTs^16,17^, which express a surface glycoprotein (gp82) that binds gastric mucin and facilitates invasion of the gastric mucosa^28,29^. Consistent with this, our infection imaging experiments showed that early acute infection was confined to the stomach. Deposition of MTs in the mouse oral cavity also caused infection, but this required a higher inoculum. Comparison with previous work using the Tula *T. cruzi* strain^30^ indicates that the oral MT infectivity of the CL Brener strain is comparatively low, even though both strains belong to the same genetic lineage (TcVI). Experimental studies suggest there are genetic factors that influence oral infectivity^31^. Nevertheless, most of the major *T. cruzi* subgroups are implicated in human oral Chagas disease cases^14,32–35^, so variability of these putative determinants can be expected to occur at the level of individual strains.

Oral transmission involving mammalian stage parasites (BTs and amastigotes) is rarely studied. A few cases of acute infection linked to consumption of infected meat from animal reservoirs have been reported^36^ and carnivory is a component of sylvatic transmission cycles^37^. In our experiments, BTs were not transmissible via the stomach. Unlike MTs, these forms are not adapted for low pH environments and do not express gp82^38^. Hoft^30^ reported a single mouse (from a total of five) that became infected after buccal inoculation with Tula strain BTs, but concluded this was probably an artefact related to mucosal tissue damage or disease. Our finding that infection occurred in 36% of healthy animals after buccal inoculation of CL Brener strain BTs shows that these parasite forms are infectious via this route, with a transmission efficiency comparable to MTs. A recent imaging study showed that naso-maxillary tissues are the main site of early parasite replication after buccal infection with tissue culture derived trypomastigotes (TCTs)^39^. In our *ex vivo* tissue analysis at the comparable time point (6 dpi), the infections in both the MT and BT buccal transmission groups were below the limit of detection. Differences in parameters such as inoculum size or parasite strain could explain this disparity, or it may reflect intrinsic differences between BTs and TCTs.

Tracking the long-term infection dynamics of chronic *T. cruzi* infections and establishing their impact on Chagas disease has only recently become possible, through the development of sufficiently sensitive imaging models^24^. We observed that over a four month course of infection, bioluminescence-inferred parasite loads in orally infected mice were very similar to those in needle-injected controls. The only notable exception was the early acute phase, 1–2 weeks post-infection, when parasite loads were lower and much less disseminated. There were no mortalities and acute symptoms were mild, generic and similar between oral and needle-injected groups. Therefore, the high mortality rates in human oral transmission cases^16^ are likely to be better explained by factors such as inoculum size, parasite genetics and lack of previous exposure to *T. cruzi* than the route of parasite entry.

The large intestine, stomach, GI mesenteric tissue and skin were the main sites of chronic parasite persistence after oral infection. These are the same sites we had previously identified for mice that were infected via i.p., i.v. and s.c. injection routes^22,23^. However, this is the first demonstration that these tissues act as infection reservoirs after transmission by a natural route that has caused high numbers of human infections. Persistence in the stomach after intra-gastric invasion indicates that primary exposure of the host to *T. cruzi* antigens in mucosal sites generates neither localised protective immunity, nor enhanced tolerance to infection. This is consistent with data showing oral infection generates broadly similar mucosal and systemic CD8^+^ T cell responses to i.p. or s.c. injections, and does not preferentially induce a “gut-homing” T cell phenotype^18^.

Repeated cycles of inflammation and tissue repair in the heart tissue of hosts with chronic *T. cruzi* infection leads to cardiac fibrosis, which is central to the pathogenesis of Chagas cardiomyopathy^24–26^. We found that orally infected mice developed cardiac fibrosis of a similar severity to needle-injected controls. Therefore, mucosal transmission does not result in long-term host-parasite interaction dynamics that are specifically more or less pathogenic than non-mucosal transmission. This has potential implications for management of chronic Chagas disease patients and the design of oral anti-*T. cruzi* vaccines.

## Methods

### Parasites, mice and infections

*T. cruzi* CL Brener (TcVI) constitutively expressing the red-shifted firefly luciferase *PpyRE9h*^23^ were used in all experiments. Infectious metacyclic trypomastigotes (MTs) and blood trypomastigotes (BTs) were generated as previously described^23^. Female BALB/c mice aged 8–12 weeks were infected with 10^4^ or 10^5^ BTs or MTs by i.p. injection of 0.2 mL, oral gavage into the stomach (intra-gastric route) of 0.2 mL, or deposition in the oral cavity using a pipette (buccal route) of 20 µL. Animal work was approved by the LSHTM Animal Welfare and Ethical Review Board and carried out under UK Home Office project licence (PPLs 70/6997 and 70/8207) in accordance with the UK Animals (Scientific Procedures) Act. BALB/c mice were purchased from Charles River (UK) and maintained under specific pathogen-free conditions in individually ventilated cages, with a 12 hour light/dark cycle and *ad libitum* food and water.

### Bioluminescence imaging

Mice were injected with 150 mg/kg d-luciferin i.p., then anaesthetized using 2.5% (v/v) gaseous isoflurane in oxygen. To measure bioluminescence, mice were placed in an IVIS Lumina II system (Caliper Life Science) and images were acquired 10–20 minutes after d-luciferin administration using LivingImage 4.3. Exposure times varied between 30 seconds and 5 minutes, depending on signal intensity. After imaging, mice were revived and returned to cages. For *ex vivo* imaging, mice were injected with 150 mg/kg d-luciferin i.p., then sacrificed by ex-sanguination under terminal anaesthesia 7 minutes later. Mice were perfused with 10 mL 0.3 mg/mL d-luciferin in PBS via the heart. Organs and tissues were transferred to culture dishes, soaked in 0.3 mg/mL d-luciferin in PBS, and then imaged in the IVIS system.

To estimate parasite burden in live mice, regions of interest (ROIs) were drawn using LivingImage v.4.3 to quantify bioluminescence expressed as total flux (photons/second) summed from dorsal and ventral images. The detection threshold for *in vivo* imaging was determined using control uninfected mice. *Ex vivo* images of tissues and organs were scored for the presence of *T. cruzi* using a detection threshold for infection foci of at least 10 contiguous bioluminescent pixels of radiance ≥3 × 10^3^ photons/second/cm^2^/sr. These criteria were established by reference to uninfected control animals.

### Histopathology

Heart samples were fixed in GlyoFixx (Thermo Scientific) for 24–72 hours, then dehydrated, cleared, and embedded in paraffin. Three micron sections were stained with haematoxylin for 8 minutes, followed by picro-sirius red for 1 hour, then dehydrated and mounted with DPX. Ten 400X magnification images of randomly selected fields covering the ventricular and atrial regions were taken on a Leica DM3000 microscope for quantitative histomorphometric analysis. The base of the heart and major vessels were excluded due to high inherent collagen content. A fibrosis index was derived by automated quantification of the proportion of tissue staining positive for collagen (red pixels), using Leica Application Suite v4.5.0. An increase in collagen content compared to uninfected controls was considered indicative of myocardial fibrosis.

### Statistics

Individual animals were used as the unit of analysis. Groups were compared using Student’s *t*-test or one-way ANOVA, with Tukey’s post-hoc correction in GraphPad Prism v.7.

### Data availability

Materials, data and associated protocols are available on request.

## Electronic supplementary material

Supplementary Figures
