# Supplementary material for: Imaging the development of chronic Chagas disease after oral transmission
Source: Sci Rep. 2018 Jul 26;8:11292. doi: 10.1038/s41598-018-29564-7 (PMC6062536; doi:10.1038/s41598-018-29564-7)
Supplement: Supplementary file 1 — Supplementary Figures [file 41598_2018_29564_MOESM1_ESM.pdf]

Supplementary Information

Imaging the development of chronic Chagas disease after oral transmission

Michael D. Lewis, Amanda F. Francisco, Shiromani Jayawardhana, Harry Langston, Martin C. Taylor, and John M. Kelly

---

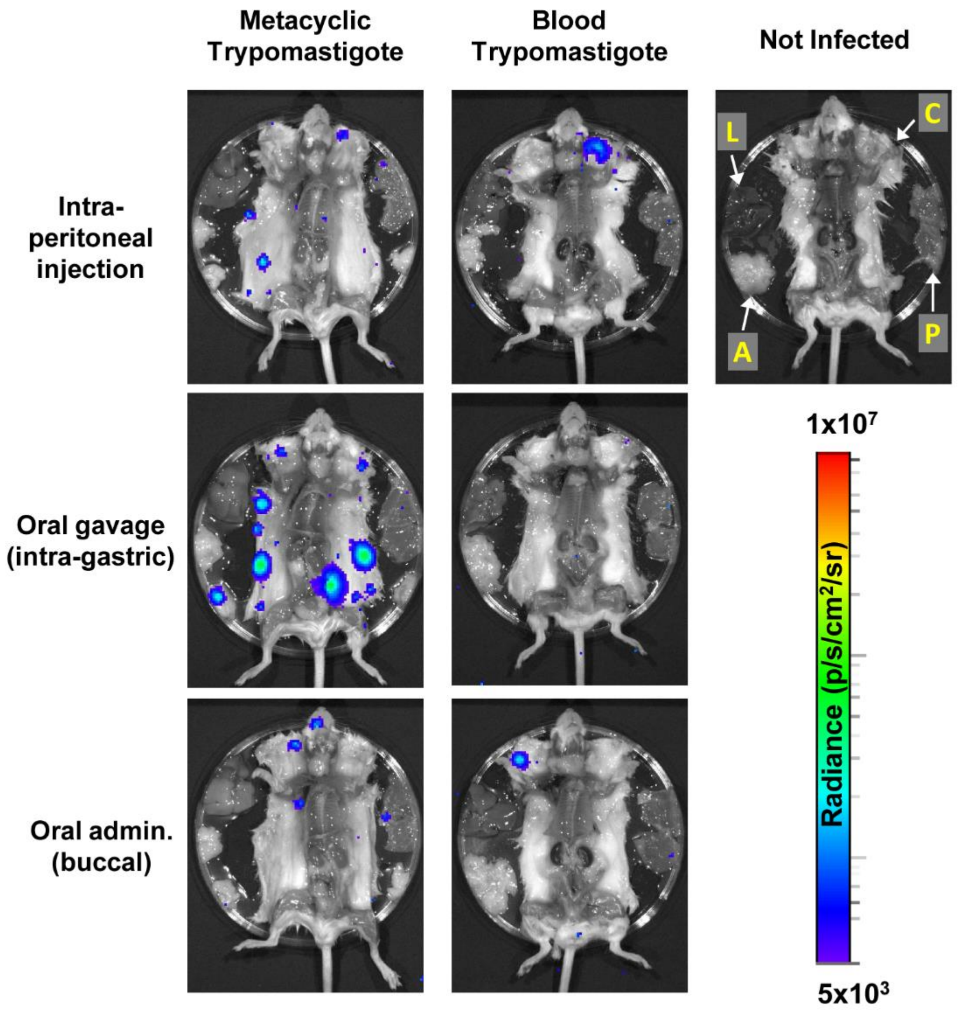

Supplementary Figure 1. Ex vivo tissue bioluminescence imaging of chronic *T. cruzi* infected mice. Mice were infected with either metacyclic or bloodstream *T. cruzi* trypomastigotes (MT, BT) by i.p. injection, oral gavage [p.o. (g)] into the stomach or deposition in the oral cavity [p.o. (b)]. Parasite distribution was assessed by bioluminescence signal in from mice at 4 months post-infection. Log-scale pseudocolour heat-maps show intensity of bioluminescence; minimum and maximum radiances are indicated. Samples arranged as labelled in the not infected image: C, carcass; L, liver; A, adipose (visceral); P, peritoneum. The principal *T. cruzi* target organs/tissues were imaged separately, data are shown in Figure 4.

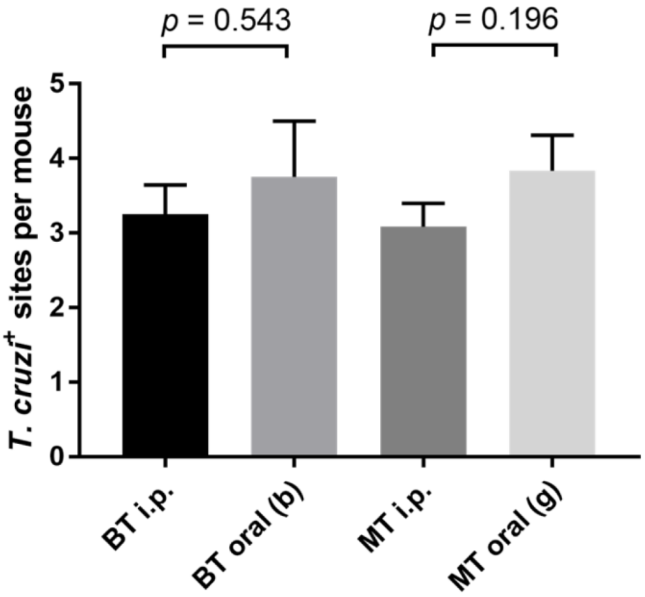

Supplementary Figure 2. Level of chronic infection dissemination. Mice were infected with either metacyclic or bloodstream *T. cruzi* trypomastigotes (MT, BT) by i.p. injection or orally by deposition into the oral cavity (b) or by gavage into the stomach (g). Ex vivo bioluminescence imaging was peformed on organs and tissues from mice at 4 months post-infection to detect parasites. Parasite distribution was assessed in the gastrointestinal tract and associated mesenteries, the heart, lung, skin, liver, spleen, peritoneum, samples of visceral adipose and skeletal muscle tissue, and the remaining carcass. Data are the means ±SEM, pooled from three experiments, compared using Student's *t*-test. BT i.p. n = 12, BT oral (b) n = 4, MT i.p. n = 12, MT oral (g) n = 6.
